# Modified Sick Neonatal Score in Predicting Outcomes of Neonates Admitted to the NICU at a Tertiary Care Center in Northern India

**DOI:** 10.7759/cureus.109349

**Published:** 2026-05-21

**Authors:** Goud Priyanka, Ekansh Rathoria, Mridula Srivastava, Utkarsh Bansal, Prerna Priyadarshini, Rohitash Lahari, Richa Rathoria

**Affiliations:** 1 Pediatrics, Hind Institute of Medical Sciences, Sitapur, IND; 2 Pediatrics, Hind Institute of Medical Sciences, Barabanki, IND; 3 Obstetrics and Gynecology, Hind Institute of Medical Sciences, Sitapur, IND

**Keywords:** critical illness, infant mortality, modified sick neonatal score, neonatal intensive care units, newborn, north india, resource-limited settings, risk assessment, tertiary care centers

## Abstract

Background

Early identification of critically ill neonates is essential for timely intervention and improved outcomes, particularly in resource-limited settings. Several neonatal illness severity scoring systems have been developed to predict mortality; however, many rely on laboratory investigations and complex calculations, limiting their applicability in low-resource health care settings. The Modified Sick Neonatal Score (MSNS) is a simple bedside clinical scoring system based on easily measurable parameters that may help predict neonatal mortality and severity of illness. This study aimed to assess the utility and predictive accuracy of the MSNS in neonates admitted to a tertiary care NICU in Northern India.

Materials and methods

This prospective observational study was conducted in the NICU of a tertiary care center in North India over 18 months. A total of 190 neonates aged ≤28 days were enrolled using systematic sampling. The MSNS was calculated at admission for each neonate. Clinical outcomes, including mortality, need for respiratory support, inotropic support, and duration of hospital stay, were recorded. Statistical analysis was performed using IBM SPSS Statistics for Windows, version 26.0 (released 2018; IBM Corp., Armonk, NY, USA). A p-value < 0.05 was considered statistically significant. Receiver operating characteristic (ROC) curve analysis was used to assess the predictive accuracy of MSNS for mortality.

Results

Out of 190 neonates, 30/190 (15.8%) died, while 160/190 (84.2%) were discharged. The mean MSNS score was significantly lower among non-survivors compared with survivors (7.87 ± 1.70 vs 10.80 ± 1.75; p < 0.001). Neonates requiring respiratory and inotropic support had significantly lower MSNS scores. The area under the ROC curve for MSNS in predicting mortality was 0.894. At a cutoff value of <10, MSNS demonstrated a sensitivity of 90.0%, a specificity of 77.5%, a positive predictive value of 42.9%, a negative predictive value of 97.6%, and an overall accuracy of 79.5% for predicting neonatal mortality.

Conclusions

The MSNS is a simple and practical bedside tool with strong predictive ability for neonatal mortality and severity of illness. Lower MSNS scores were significantly associated with mortality, need for respiratory support, and inotropic requirement among admitted neonates. The MSNS demonstrated high sensitivity and negative predictive value, supporting its reliability for the early identification of high-risk neonates. Its ease of application without laboratory investigations makes it particularly valuable for timely clinical decision-making in resource-limited settings.

## Introduction

The neonatal period, defined as the first 28 days of life, represents the most critical phase for child survival, with an estimated 2.3 million neonatal deaths worldwide in 2023, accounting for nearly 6,300 deaths each day [1]. Neonatal mortality is nearly nine times higher in low-income countries than in high-income settings [2]. Global and national initiatives, including the Sustainable Development Goals (SDG) and India’s Newborn Action Plan 2014, emphasize reduction in neonatal mortality as a key priority [3].

India alone contributes nearly one-fourth of global neonatal deaths, with a disproportionate burden borne by rural and semi-urban regions [4]. The National Family Health Survey (NFHS-5) reports an Indian neonatal mortality rate (NMR) of 24.9 per 1,000 live births, with prematurity, sepsis, and birth asphyxia accounting for most neonatal deaths [5]. According to recent UNICEF estimates, India’s NMR declined from 28 per 1,000 live births in 2013 to 17 per 1,000 live births in 2023 [6].

Regional disparities further highlight the burden at the local level. In Sitapur district, the NMR and under-five mortality rate (U5MR) are 60.64 and 95.95 per 1,000 live births, respectively, which are considerably higher than the recommended SDG global targets (NMR ≤12 and U5MR ≤25 per 1,000 live births, respectively) [7]. These figures underscore the urgent need for early identification and effective management of high-risk neonates.

Early identification of critically ill neonates using rapid, objective severity-assessment tools at admission is essential for guiding timely clinical decisions, optimizing resources, and improving survival [8]. Several neonatal illness severity scoring systems, including the Score for Neonatal Acute Physiology (SNAP), Score for Neonatal Acute Physiology with Perinatal Extension II (SNAPPE-II), Clinical Risk Index for Babies (CRIB), and the Neonatal Therapeutic Intervention Scoring System (NTISS), have been developed to predict outcomes in neonates admitted to the NICU [9,10]. Although these scoring systems show good predictive accuracy, their reliance on laboratory tests, invasive monitoring, and complex calculations limits their applicability in limited infrastructure settings, underscoring the need for simpler bedside tools based on easily measurable clinical parameters with reliable prognostic value [11,12].

To overcome these limitations, Mansoor et al. developed the Modified Sick Neonatal Score (MSNS) as a simplified adaptation of the validated Sick Neonatal Score for use in resource-limited settings [13]. It incorporates eight routinely assessed variables, namely respiratory effort, heart rate, axillary temperature, capillary refill time, random blood sugar, oxygen saturation, gestational age, and birth weight, allowing rapid assessment without the need for sophisticated equipment [14]. Preliminary studies have shown encouraging results regarding its ability to predict neonatal mortality and adverse outcomes, particularly in district hospitals and low-resource NICUs [15].

Despite its potential advantages, validation of the MSNS in diverse Indian clinical settings, particularly rural tertiary care NICUs managing a high burden of critically ill and preterm neonates, remains limited [16]. This prospective study was undertaken to evaluate the predictive accuracy of MSNS in neonates admitted to a rural tertiary care NICU, assess its association with neonatal mortality and clinical outcomes, and validate its utility as a simple, rapid, and cost-effective prognostic tool in resource-limited settings.

## Materials and methods

Study design and setting

This prospective observational study was conducted in the NICU of the Department of Pediatrics at Hind Institute of Medical Sciences, Sitapur, over a period of 18 months from August 2024 to January 2026. The NICU is a tertiary care referral center catering to both inborn and outborn neonates from surrounding rural areas.

Study population

All neonates admitted to the NICU during the study period were screened for eligibility based on predefined inclusion and exclusion criteria. Neonates aged ≤28 days of either sex with a gestational age of ≥28 weeks were included in the study. Neonates with major congenital anomalies incompatible with life, acute surgical emergencies, or syndromic conditions were excluded. Neonates who were discharged against medical advice or referred to other centers before completion of outcome assessment were excluded from the final analysis.

Sample size

The sample size was calculated using the formula, based on an SD of 1.753 from a previous study, with a 95% confidence level and an absolute precision of 0.25 [12]. The minimum required sample size was 189, which was rounded to 190.

Data collection and MSNS assessment

After obtaining consent, detailed maternal, demographic, and clinical information was collected using a predesigned structured pro forma, including variables such as sex, residence, mode of delivery, gestational age, birth weight, and primary diagnosis at admission. The MSNS, originally described by Mansoor et al., is an open-access clinical scoring system that was calculated for each neonate at the time of initial clinical evaluation immediately upon NICU admission, before the initiation of definitive therapeutic interventions, using standardized criteria to ensure uniformity and avoid treatment-related alterations in clinical parameters, and was not used as a serial monitoring tool (Table 1) [13]. The score comprises eight parameters, each assigned a score of 0, 1, or 2, with a total possible score ranging from 0 to 16; lower scores indicate greater severity of illness.

**Table 1:** MSNS parameters and scoring criteria MSNS, Modified Sick Neonatal Score Source: Mansoor et al. (2019) [13]; Creative Commons Attribution (CC BY) license

Parameter	Score 0	Score 1	Score 2
Respiratory effort	Apnea or grunt	Tachypnea (respiratory rate >60/minute) with or without retractions	Normal (respiratory rate 40-60/minute)
Heart rate (per minute)	Bradycardia (<100/minute) or asystole	Tachycardia (>160/minute)	Normal (100-160/minute)
Axillary temperature (°C)	<36	36-36.5	36.5-37.5
Capillary refill time (seconds)	>5	3-5	<3
Random blood sugar (mg/dL)	<40	40-60	>60
SpO₂ in room air (%)	<85	85-92	>92
Gestational age (in weeks)	<32 weeks	32-36 weeks + 6/7 days	≥37 weeks
Birth weight (g)	<1500	1500-2499	≥2500

Outcome measures

All neonates were followed until a definitive outcome, categorized as either survival (discharged alive) or mortality (death during the hospital stay). Secondary outcomes assessed included the requirement for respiratory support (both invasive and noninvasive), the need for inotropic support, and the duration of hospital stay.

Statistical analysis

Data were entered into Microsoft Excel (Microsoft Corporation, Redmond, WA, USA) and analyzed using IBM SPSS Statistics for Windows, version 26.0 (released 2018; IBM Corp., Armonk, NY, USA). Continuous variables were expressed as mean ± SD, while categorical variables were presented as frequencies and percentages. Group comparisons were performed using the independent t-test for continuous variables and the chi-square test or Fisher’s exact test for categorical variables, as appropriate. Receiver operating characteristic (ROC) curve analysis was performed to evaluate the predictive accuracy of MSNS for mortality, and the area under the ROC curve (AUC) was calculated. A p-value < 0.05 was considered statistically significant.

Ethical approval

The study was conducted after obtaining approval from the Institutional Ethics Committee of Hind Institute of Medical Sciences, Sitapur (IHEC-HIMSA/MD/MS-23/RD-36/07-24). Written informed consent was obtained from the parents or legal guardians of all participants, and confidentiality and anonymity of patient data were strictly maintained throughout the study.

## Results

Study participant flow

**Figure 1:**
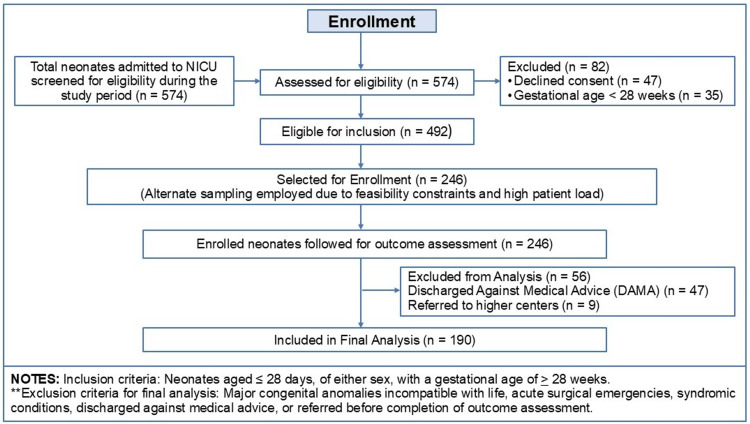
Flow diagram of study participant enrollment

A total of 574 neonates were admitted during the study period. Of these, 47 parents declined consent, and 35 neonates with a gestational age of less than 28 weeks were excluded. The remaining 492 neonates were eligible for inclusion. Due to operational constraints, including high patient load and limited availability of trained personnel to perform standardized MSNS assessments at all hours of admission, systematic (alternate) sampling was employed using a predefined approach, wherein enrollment began with the first eligible neonate admitted during the study period and subsequently included every alternate eligible admission to minimize selection bias, resulting in the enrollment of 246 neonates. Among these, 47 neonates were discharged against medical advice, and nine were referred to higher centers before completion of outcome assessment, resulting in a final sample of 190 neonates for analysis (Figure 1).

The baseline demographic and perinatal characteristics of the study population are summarized in Table 2. The study population (n = 190) consisted primarily of male neonates (121/190, 63.7%) and those residing in rural areas (153/190, 80.5%). Perinatal characteristics revealed a high prevalence of high-risk factors, with 80% (152/190) of births occurring via lower-segment cesarean section and the majority of the cohort being preterm (144/190, 75.8%). Regarding birth weight, 42.1% (80/190) of neonates weighed between 1500 and 2499 g, while 31.1% (59/190) were classified as very low birth weight (<1500 g), highlighting the clinical vulnerability of the participants recruited for MSNS evaluation.

**Table 2:** Baseline demographic and perinatal characteristics of study participants (n = 190)

Variable	Category	Frequency (n)	Percentage (%)
Gender	Male	121	63.7
	Female	69	36.3
Residence	Rural	153	80.5
	Urban	37	19.5
Mode of delivery	Lower-segment cesarean section	152	80
	Vaginal delivery	38	20
Gestational age	Preterm	144	75.8
	Term	46	24.2
Birth weight	<1500 g	59	31.1
	1500-2499 g	80	42.1
	≥2500 g	51	26.8

Analysis of individual MSNS parameters revealed that physiological and demographic factors significantly influenced neonatal outcomes (Table 3). Out of the 190 neonates included in the study, 30/190 (15.8%) expired during the hospital stay, while 160/190 (84.2%) were discharged. Gestational age and birth weight were the strongest predictors of mortality (p < 0.001), with 83.3% (25/30) and 73.3% (22/30) of deaths occurring in the lowest scoring categories, respectively. Among clinical signs, capillary refill time (p = 0.002), heart rate (p = 0.010), and oxygen saturation (p = 0.011) showed statistically significant associations with mortality. Conversely, respiratory effort (p = 0.137), axillary temperature (p = 0.732), and random blood sugar (p = 0.704) did not demonstrate significant independent associations with death in this cohort. These findings indicate that while the total MSNS is a robust predictor, its accuracy is driven primarily by birth weight, maturity, and specific markers of perfusion and oxygenation.

**Table 3:** Association of MSNS parameters with neonatal outcome (n = 190) Data are presented as n (%); p-value < 0.05 was considered statistically significant. FET, Fisher’s exact test; MSNS, Modified Sick Neonatal Score

Parameter	Score	Death (n = 30)	Discharge (n = 160)	Statistical test	p-value
Respiratory effort	0	23 (76.6)	90 (56.3)	FET = 4.048	0.137
	1	5 (16.7)	45 (28.1)		
	2	2 (6.7)	25 (15.6)		
Heart rate	0	5 (16.7)	4 (2.5)	FET = 8.763	0.01
	1	8 (26.7)	43 (26.9)		
	2	17 (56.6)	113 (70.6)		
Axillary temperature	0	2 (6.7)	6 (3.7)	FET = 0.919	0.732
	1	17 (56.6)	90 (56.3)		
	2	11 (36.7)	64 (40.0)		
Capillary refill time	0	1 (3.3)	0 (0.0)	FET = 11.670	0.002
	1	10 (33.3)	20 (12.5)		
	2	19 (63.4)	140 (87.5)		
Random blood sugar	0	2 (6.7)	14 (8.8)	FET = 0.838	0.704
	1	3 (10.0)	10 (6.2)		
	2	25 (83.3)	136 (85.0)		
Oxygen saturation in room air	0	5 (16.7)	16 (10.0)	χ² = 9.134	0.011
	1	20 (66.6)	70 (43.7)		
	2	5 (16.7)	74 (46.3)		
Gestational age	0	25 (83.3)	32 (20.0)	FET = 45.419	<0.001
	1	5 (16.7)	82 (51.2)		
	2	0 (0.0)	46 (28.8)		
Birth weight	0	22 (73.3)	37 (23.1)	FET = 27.077	<0.001
	1	6 (20.0)	74 (46.3)		
	2	2 (6.7)	49 (30.6)		

Out of 190 neonates, 30/190 (15.8%) died during hospitalization, while 160/190 (84.2%) were discharged (Table 4). Respiratory support was required in 155/190 (81.6%) neonates, whereas 103/190 (54.2%) required inotropic support. Among the 155 neonates requiring respiratory support, 91/155 (58.7%) received invasive mechanical ventilation, while 64/155 (41.3%) were managed with noninvasive ventilation using either continuous positive airway pressure or high-flow nasal cannula. The duration of hospital stay was <7 days in 75/190 (39.5%) neonates and ≥7 days in 115/190 (60.5%).

**Table 4:** Relationship between MSNS and clinical outcomes in neonates (n = 190) Statistical test used: independent t-test. Data are presented as n (%) and mean ± SD; p-value < 0.05 was considered statistically significant. MSNS, Modified Sick Neonatal Score

Variables	Category	Frequency, n (%)	MSNS score (mean ± SD)	t-test value	p-value
Outcome	Death	30 (15.8)	7.87 ± 1.70	-8.459	<0.001
	Discharge	160 (84.2)	10.80 ± 1.75		
Need for respiratory support	No	35 (18.4)	10.97 ± 2.03	2.052	0.042
	Yes	155 (81.6)	10.19 ± 2.0		
Need for inotropes	No	87 (45.8)	11.94 ± 1.09	14.409	<0.001
	Yes	103 (54.2)	8.98 ± 1.63		
Duration of hospital stay	<7 days	75 (39.5)	9.51 ± 2.05	-4.779	<0.001
	≥7 days	115 (60.5)	10.88 ± 1.85		

The MSNS showed a strong association with neonatal clinical outcomes (Table 4). The total mean MSNS score of the study population was 10.34 ± 2.042. Neonates who died had significantly lower mean MSNS scores (7.87 ± 1.70) than those who survived (10.80 ± 1.75; p < 0.001). Lower scores also significantly correlated with the requirement for inotropic support (p < 0.001) and respiratory support (p = 0.042). Regarding mortality distribution, 63.3% (19/30) of deaths occurred within the first seven days of admission, while 36.7% (11/30) occurred after seven days. A hospital stay of <7 days was associated with lower mean scores (9.51 ± 2.05) compared with longer stays (10.88 ± 1.85; p < 0.001), reflecting higher initial acuity.

**Figure 2:**
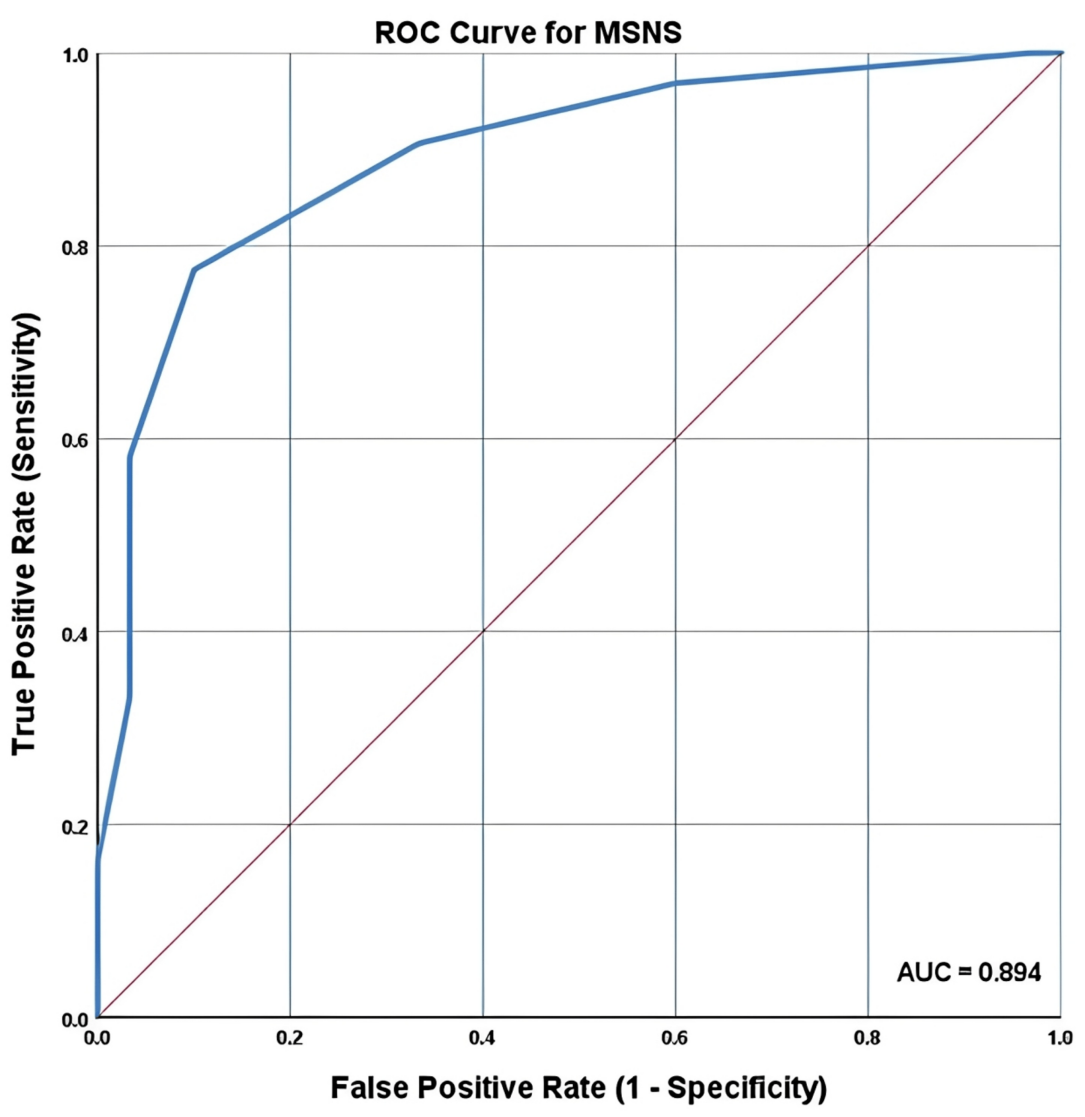
ROC curve showing the diagnostic performance of MSNS in predicting neonatal mortality AUC, area under the ROC curve; MSNS, Modified Sick Neonatal Score; ROC, receiver operating characteristic

The MSNS demonstrated excellent diagnostic performance in predicting neonatal mortality (Table 5). At a cutoff value of <10, the score achieved a sensitivity of 90.0% and a specificity of 77.5%. The negative predictive value (NPV) was remarkably high at 97.6%, while the positive predictive value (PPV) was 42.9%. The overall diagnostic accuracy of the tool was 79.5%. ROC curve analysis further confirmed the robustness of the MSNS, with an AUC of 0.894 (95% CI: 0.816-0.971) (Figure 2).

**Table 5:** Diagnostic performance of MSNS at a cutoff value of <10 for the prediction of neonatal mortality Data are presented as percentages (%). Values represent the diagnostic performance of MSNS at a cutoff value of <10. MSNS, Modified Sick Neonatal Score; NPV, negative predictive value; PPV, positive predictive value

Parameter	Value (%)	95% CI (%)
Sensitivity	90	74.4-96.5
Specificity	77.5	70.4-83.3
PPV	42.9	31.4-55.1
NPV	97.6	93.3-99.2
Accuracy	79.5	73.2-84.6

## Discussion

The present prospective observational study evaluated the utility of the MSNS as a predictor of mortality and clinical outcomes among neonates admitted to a tertiary care NICU in a rural setting. Our findings demonstrate that MSNS is a simple, reliable, and effective bedside tool with strong predictive ability for neonatal mortality, making it particularly valuable in resource-limited settings.

The overall NMR observed in this study was 15.8% (30/190), which is comparable to previously reported rates from similar tertiary care centers in developing countries. Studies by Meshram and Merchant and Reddy et al. have reported mortality rates ranging from 12% to 20% among NICU admissions [14,15]. The high proportion of preterm and low birth weight neonates in our study cohort likely contributed to this mortality pattern, as these factors are well-established determinants of neonatal outcomes [2]. In addition, maternal factors such as inadequate antenatal care and maternal anemia have been shown to significantly influence neonatal morbidity and mortality, especially in rural populations of this region, further contributing to adverse outcomes [17,18].

A major finding of our study was that the mean MSNS score was significantly lower among non-survivors (7.87 ± 1.70) compared with survivors (10.80 ± 1.75), with a highly significant difference (p < 0.001). This is consistent with earlier studies validating the prognostic utility of MSNS. Twanbasu et al. and Padar et al. also reported significantly lower scores among neonates who expired, confirming the inverse relationship between MSNS and illness severity [12,19]. Similarly, Jayasheel et al. demonstrated that lower MSNS scores are strongly associated with increased risk of mortality in neonates [11]. These findings reinforce that MSNS is a robust indicator of neonatal clinical status.

Among the individual components of MSNS, gestational age and birth weight emerged as the most significant predictors of mortality, with the majority of deaths occurring in neonates with the lowest scores for these parameters. This is in agreement with existing literature and previous MSNS-based studies, where prematurity and low birth weight consistently demonstrated strong associations with adverse outcomes [14,16]. Additionally, clinical parameters such as capillary refill time, heart rate, and oxygen saturation showed significant correlations with mortality, emphasizing the importance of perfusion and oxygenation in critically ill neonates. Similar observations have been reported by Shivaramakrishnababji et al. [16]. These findings indicate that while the total MSNS is a robust predictor, its accuracy is driven primarily by birth weight, maturity, and specific markers of perfusion and oxygenation.

The lack of statistically significant association of certain physiological parameters, such as respiratory effort, axillary temperature, and random blood sugar, with mortality in our study may be explained by several factors. Early stabilization practices, including maintenance of the neonatal “warm chain” and early correction of hypoglycemia and initial respiratory support, may have reduced the severity of abnormalities present at admission. In addition, the study cohort included a high proportion of preterm and low birth weight neonates, which are well-established major predictors of neonatal mortality and may have attenuated the apparent independent contribution of individual physiological variables [2,9]. These findings are consistent with the original MSNS study by Mansoor et al., which emphasized that the prognostic strength of MSNS lies in its multidimensional composite structure rather than in the isolated predictive value of individual parameters [13].

The association between MSNS and clinical interventions further underscores its clinical relevance. Neonates requiring respiratory and inotropic support had significantly lower mean MSNS scores, indicating greater severity of illness. Respiratory support included both invasive and noninvasive modes, and a substantial proportion of neonates required such support, reflecting the high acuity of cases managed at this center. Similar findings have been reported in previous studies, where lower MSNS scores were associated with an increased need for intensive care interventions [15,19]. The higher proportion of neonates requiring respiratory support compared with inotropic support likely reflects the predominance of respiratory distress in the study population, with fewer neonates developing hemodynamic instability requiring inotropes.

An interesting finding in our study was that neonates with shorter hospital stays (<7 days) had significantly lower MSNS scores compared with those with longer stays. This apparent paradox is clarified by the study's mortality distribution: 19 of the 30 total deaths (63.3%) occurred within this initial seven-day period. This suggests that the shorter duration of hospitalization in these cases was a consequence of early mortality among critically ill neonates rather than rapid clinical recovery. Similar patterns have been described in studies evaluating neonatal severity scores, where early deaths contribute to shorter durations of hospitalization among the sickest patients [9,20].

The diagnostic performance of MSNS in predicting neonatal mortality was found to be excellent in our study, with high sensitivity, specificity, and AUC. These findings are comparable to those reported in previous studies, which have demonstrated the reliability of MSNS as a prognostic tool [12,14]. The high NPV observed in our study is particularly important in clinical practice, as it helps identify neonates who are less likely to have adverse outcomes, thereby aiding in efficient resource allocation.

Systematic reviews have further emphasized the importance of simple and effective neonatal scoring systems in predicting outcomes. Aluvaala et al. highlighted that simplified clinical scoring systems are particularly useful in low-resource settings for predicting mortality and optimizing resource utilization [21]. Additionally, studies evaluating established scoring systems such as SNAP-II and SNAPPE-II have demonstrated good predictive ability but are limited by their dependence on laboratory parameters and complexity [10,22]. Mesquita Ramirez et al. reported that while these scores are accurate, their applicability may be constrained in resource-limited settings [22].

Unlike SNAP-II and SNAPPE-II, which require laboratory-based investigations, including arterial blood gas analysis and serum biochemical measurements, as well as more complex calculations, the MSNS relies entirely on simple bedside clinical parameters without requiring laboratory investigations [9,13]. Owing to its rapid bedside applicability, lower complexity, and minimal resource requirements, MSNS is particularly suitable for early risk stratification in high-volume NICUs and resource-limited healthcare settings where access to advanced investigations and monitoring may be limited [9,21].

Furthermore, studies conducted in resource-limited NICUs, such as that by Shrestha et al., have emphasized the importance of early and simple predictors of neonatal mortality, particularly in settings where advanced monitoring and investigations are not readily available [20]. These findings align with the results of our study and support the role of MSNS as a practical tool for early risk stratification.

While interventions such as kangaroo mother care have been shown to improve neonatal outcomes, especially among stable low birth weight infants, they serve as complementary strategies in similar neonatal populations from the same region as our study [23]. Additionally, studies have highlighted the role of maternal and neonatal factors, such as vitamin D status at birth, in influencing neonatal health outcomes, underscoring the multifactorial nature of neonatal morbidity within the same regional context [24]. Early identification of high-risk neonates using tools like MSNS remains crucial for timely intervention and optimal management.

Overall, the findings of this study suggest that MSNS is a simple, cost-effective, and reliable scoring system that can be easily implemented in routine clinical practice. Its ability to predict mortality and need for intensive care interventions makes it particularly valuable in resource-constrained settings. This study adds to the existing literature by validating MSNS as an effective prognostic tool in a rural North Indian NICU setting.

Limitations

This study has certain limitations. Being a single-center study conducted in a rural tertiary care hospital, the findings may not be generalizable to all settings. The sample size, although adequate, may limit subgroup analysis. MSNS was assessed only at admission, and serial assessment was not performed to evaluate dynamic changes in clinical condition. Additionally, comparison with other established scoring systems, such as SNAP-II or CRIB, was not undertaken. Although MSNS is primarily an admission-based score, future multicentric studies with larger sample sizes and comparative analyses may further explore its role in serial assessment and help validate and extend these findings.

Despite these limitations, a key strength of this study is the prospective design and use of a simple, bedside-applicable scoring system in a resource-limited setting, which enhances its clinical applicability in similar healthcare settings.

## Conclusions

The MSNS is a simple, rapid, and reliable bedside tool for assessing illness severity and predicting outcomes in neonates admitted to the NICU. In the present study, lower MSNS scores were significantly associated with higher mortality, increased need for respiratory support and inotropic support, and greater illness severity at admission. The score demonstrated excellent diagnostic performance, with high sensitivity, specificity, and NPV for predicting neonatal mortality. As MSNS relies solely on easily measurable clinical parameters without requiring laboratory investigations, it is particularly suitable for resource-limited settings. Its use can facilitate early risk stratification, prompt clinical decision-making, and efficient allocation of healthcare resources. Overall, MSNS is an effective and practical prognostic tool that can contribute to improved neonatal outcomes, especially in rural and low-resource healthcare settings.
